# A Simple Wireless Sensor Node System for Electricity Monitoring Applications: Design, Integration, and Testing with Different Piezoelectric Energy Harvesters †

**DOI:** 10.3390/s18113733

**Published:** 2018-11-02

**Authors:** Zongxian Yang, Sid Zarabi, Egon Fernandes, Maria-Isabel Rua-Taborda, Hélène Debéda, Armaghan Salehian, David Nairn, Lan Wei

**Affiliations:** 1 Department of Electrical and Computer Engineering, University of Waterloo, Waterloo, ON N2L 3G1, Canada; zongxian.yang@uwaterloo.ca (Z.Y.); ssfarshc@uwaterloo.ca (S.Z.); nairn@uwaterloo.ca (D.N.); 2 Department of Mechanical and Mechatronics Engineering, University of Waterloo, Waterloo, ON N2L 3G1, Canada; e22ferna@uwaterloo.ca (E.F.); salehian@uwaterloo.ca (A.S.); 3 IMS Laboratory, University of Bordeaux, 33400 Talence, France; maria-isabel.rua-taborda@ims-bordeaux.fr (M.-I.R.-T.); helene.debeda@ims-bordeaux.fr (H.D.)

**Keywords:** energy management, self-contained system, electricity monitoring, wireless sensor node, power conditioning circuit, energy harvester, PZT, screen-printing, dynamic duty cycle

## Abstract

Real time electricity monitoring is critical to enable intelligent and customized energy management for users in residential, educational, and commercial buildings. This paper presents the design, integration, and testing of a simple, self-contained, low-power, non-invasive system at low cost applicable for such purpose. The system is powered by piezoelectric energy harvesters (EHs) based on PZT and includes a microcontroller unit (MCU) and a central hub. Real-time information regarding the electricity consumption is measured and communicated by the system, which ultimately offers a dependable and promising solution as a wireless sensor node. The dynamic power management ensures the system to work with different types of PZT EHs at a wide range of input power. Thus, the system is robust against fluctuation of the current in the electricity grid and requires minimum adjustment if EH unit requires exchange or upgrade. Experimental results demonstrate that this unit is in a position to read and transmit 60 Hz alternating current (AC) sensor signals with a high accuracy no less than 91.4%. The system is able to achieve an operation duty cycle from <1 min up to 18 min when the current in an electric wire varies from 7.6 A to 30 A, depending on the characteristics of different EHs and intensity of current being monitored.

## 1. Introduction

In contemporary urban growth and development, the problem of extensive energy utilization in residential, educational, and commercial buildings has become crucial and urgent. About one third of total civil energy is consumed in buildings at present around the world [1]. The growing concern over enhancing the efficiency of energy consumption within residential, educational, and commercial buildings [2] has therefore attracted keen research and industry interests in developing novel intelligent power management systems based on sensors and actuators [3,4]. While an electric meter provides the utility company with information regarding the total energy consumption, no information is provided to the consumers with respect to the energy consumed by individual appliances [5]. Such information could enable consumers to better control and manage their energy usage, achieving a reduced overall energy consumption [6]. By using the emerging smart technology, the outdated electric distribution network within buildings can be replaced with automated and smart systems to increase the power consumption efficiency and optimize the power distribution [7]. Among these new technologies, wireless sensor networks (WSNs) have been widely emphasized because of their flexible and decent way to establish an intelligent power management system [8,9]. This system is able to gather, analyze, and transmit the sensory information regarding the power information, providing better efficiency and power management. Not only in continuous environmental monitoring [10,11,12] including widespread air quality monitoring and water quality monitoring, WSNs have also received increasing attentions for other various applications, such as animal tracking [13,14] and health monitoring of civil structures [15,16,17]. The applications of WSNs in different fields are based on abundant state-of-the-art sensors adopted for monitoring systems [18], human-machine interactive systems [19], wearable electronic systems [20], etc. However, WSNs are still facing some inherent challenges such as slow data transmission speed, high power consumption, insufficient data storage capacity, narrow wireless communication bandwidth, and short life time [21,22,23]. Such limitations ahead extensively restrict the development and popularity of modern WSN systems. Therefore, developing low cost, self-contained, and non-invasive wireless sensor nodes has become a major challenge to establish such infrastructure. In this work, integrated with our piezoelectric (based on PZT) energy harvesters (EHs) and power conditioning circuitry with dynamic power management scheme, the proposed system is able to solve some of the existed critical issues in order to design and develop a simple, low-cost, self-powered, robust, and non-invasive WSN for electricity monitoring applications. Tested with two different kinds of EHs in our experiments under various conditions, the robustness of the WSN system is verified. We believe that other available harvesters, sensor devices, and wireless communication units are able to be conveniently integrated with our system as a result of its increased flexibility and robustness. In particular, this allows minimum efforts and cost for potential replacement and upgrade of the harvester and sensor units. Furthermore, the design approach introduced in this paper provides a systematic approach that is easily extended to other similar applications.

The rest of the paper is organized as follows: Section 2 describes the major components of the system and their corresponding functionalities. Section 3 introduces our methodology in circuit design and embedded software development. Section 4 presents the experimental implementation and testing method to validate the system’s performance. Results and comparisons are discussed in Section 5 including analysis with respect to performance, error, transmission duty cycle, and reliability. Finally, Section 6 concludes the work.

## 2. System Description

**Figure 1:**
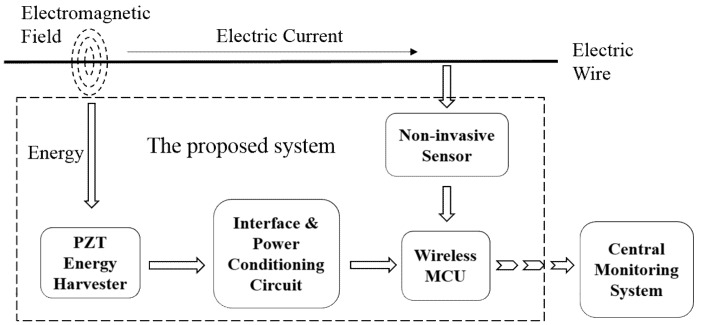
Overall schematic of the self-contained WSN monitoring system independent of battery.

The schematic of the self-contained system shown in Figure 1 consists of a PZT-based piezoelectric EH, a power conditioning circuit, a non-invasive piezoelectric-based current sensor, a wireless microcontroller unit (MCU), and a wireless transceiver in central monitoring system. Each component plays an indispensable role in the operation of the unit. They are selected considering the factors such as power consumption, size, cost, and reliability so that each of them operates and functions most effectively. The WSN system is powered by the energy harvested from the electromagnetic field across the electric wires, thus independent of battery supply. Furthermore, interfaces and the power conditioning circuitry are designed in a flexible way to boost the adjustability of the system. In this way, various EHs and sensor devices can be integrated with the system as well. In this work, we realize and test the system with two different PZT EHs in order to validate its functionality, flexibility and robustness. The wide range of input power provided by the two EHs indicates the potential of the WSN to work with other low-power, state-of-the-art devices.

### 2.1. Piezoelectric Energy Harvesters Based on PZT

In the case of non-invasive wireless sensor nodes for electricity monitoring applications, the electromagnetic energy is the dominant and most accessible source available to the EH [24]. Through the PZT-based piezoelectric harvester with permanent magnets, the ambient electromagnetic energy generated from the current-carrying wire is finally converted into electrical energy in a non-invasive manner after an intermediate form of mechanical vibrational energy. The transformed electric energy can be stored in the storage unit (e.g., super-capacitor) and provides the required energy for the WSN. This eliminates the need of external power supply such as batteries.

**Figure 2:**
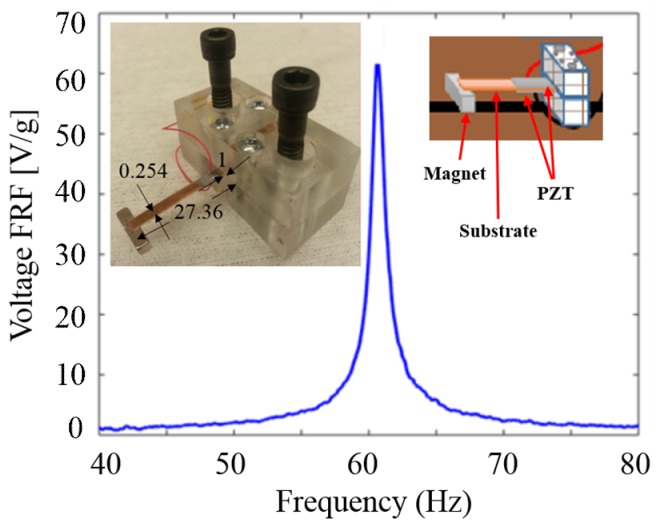
Measured voltage frequency response function of the simple PZT-based cantilever beam EH (EH1 shown in the inset (dimension unit in mm)) with the peak voltage at 60.2 Hz resonance frequency which matches the North American 60 Hz power line frequency.

A number of structures have been reported for PZT-based EHs. One of the most popular configurations is the cantilever beam structure [25,26,27]. In our work, a harvester with bulk PZT and permanent magnet tip mass is firstly used with the structure of a simple piezoelectric bimorph cantilever beam. In the experiment, the distance between our cantilever EH (EH1) and the electric wire that carries a 60-Hz AC current is 6 mm. The surrounding electromagnetic energy from the field across the wire is collected by the harvester non-invasively. The voltage Frequency Response Function (FRF) of EH1 is presented as in Figure 2 with the peak voltage at 60.2 Hz resonance frequency which matches the North American 60 Hz power line frequency.

**Figure 3:**
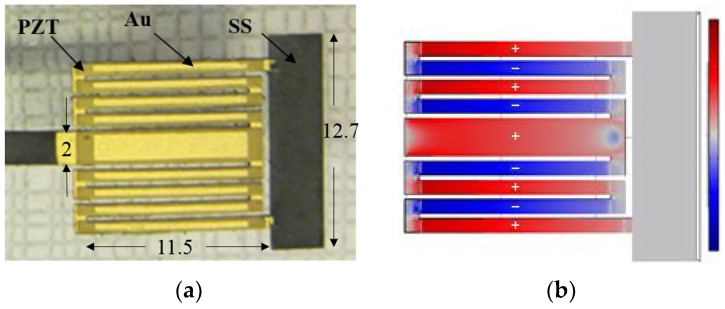
(**a**) Fabricated printed PZT energy harvester with Au electrodes (EH2 (dimension unit in mm)); (**b**) Strain contour plot showing tension (red) and compression (blue) in EH2. The structure of meandering PZT-based piezoelectric energy harvester (EH2) with a centrally-supported meandering geometry fabricated using screen-printing technology [28].

**Figure 4:**
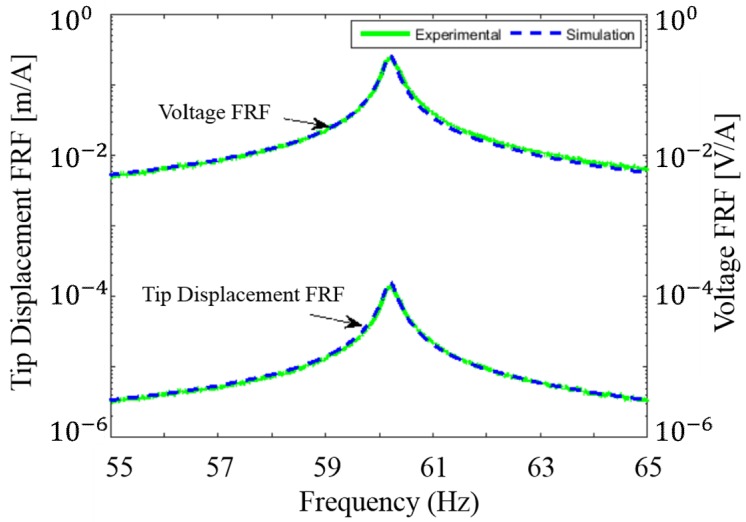
Tip displacement and voltage Frequency Response Function of the EH2 with the peak voltage at 60.3 Hz resonance frequency [28].

The second PZT-based piezoelectric EH (EH2) shown in Figure 3a for further system testing is more advanced and fabricated using screen-printing technology on a stainless steel (SS) substrate. The design employs a so-called meandering geometry which is centrally-supported [28]. This structure is known to be capable of achieving better power efficiency than that of a zigzag structure owing to the reduced torsional mode effects in the clamped beam [29]. As presented in Figure 3b, during the cyclic motion, each individual beam experiences either tension (shown in red) or compression (shown in blue) along its entire length. The adjacent beams experience regularly alternating strains to generate periodic alternating output currents. Moreover, by using the novel screen-printing techniques, one is able to obtain more advantages such as relatively lower investment costs for both equipment and materials compared to others and high volume manufacturing with automatic process line. The experimental and simulated (using COMSOL model) tip displacement and open-circuit voltage FRFs are shown in Figure 4 when the distance between EH2 and the electric wire with 1 A AC current is approximately 12.5 mm. The experimental resonant frequency is shown to be 60.3 Hz, matching that of the North American power grid. The simulation shows just a negligible error as well where the COMSOL model gives us a prediction of the resonant frequency to be 60.2 Hz. During the subsequent test of the system, this EH harvests energy from the electric wire at a distance of 4.5 mm (the minimum) for a higher output power.

**Figure 5:**
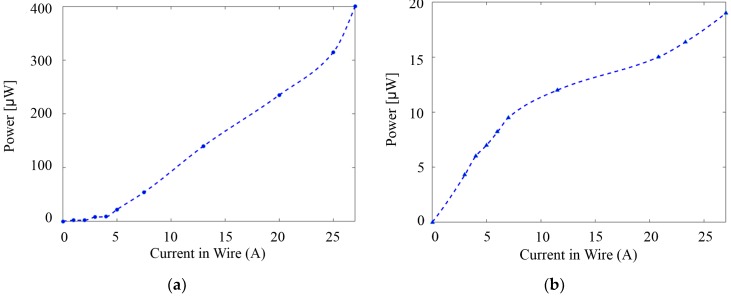
(**a**) Power output of EH1; (**b**) Power output of EH2. Measured RMS power output of two harvesters with regard to the current passing through the conducting wire. (The distance between EH1 and the wire is 6 mm and 4.5 mm for EH2 for a higher output power) EH2 has much lower output power than EH1 as a result of screen-printed PZT versus bulk PZT.

The average power output and performance of a vibrational EH are determined by a variety of factors such as size, structure, input stimulus, operating frequency, operating environment, and the impedance of the interface circuitry responsible for energy regulation and storage [30]. This power output should be able to fulfil the power requirements of electronic devices within the integrated system. The intensity of power output from one designated electromagnetic PZT EH in this work varies mainly because of the input stimulus, i.e., its distance from the conducting wire and the intensity of current carried by the wire. Figure 5 presents the measured RMS power output of EH1 and EH2 with regard to the current. EH2 has much lower output power since it uses screen-printing PZT, which results in a thinner layer of PZT with lower densification. However, screen-printing technique has major advantages of low cost, possibility to add electrodes with different shapes and suitable for high volume production, as mentioned in previous section.

### 2.2. Interface and Power Conditioning Circuit

The AC signal across the load connected to the EH has to be processed (rectified, regulated, and conditioned) before it goes to the load circuitry to power the WSN system. The rectified current charges a unit for energy storage purpose such as a battery which is rechargeable or a widely-used super capacitor which can be subsequently conditioned to drive the integrated electronic devices. Some important and common interface circuitries intended for power conditioning have been explored and available in the literature [31,32,33] within energy harvesting systems. Popular circuities vary from simplest bridge rectifiers in simple and low-cost applications to complicated active converter circuits adopted in the design of intelligent control systems for high power density and low noise [34]. Different power conditioning technologies are considered regarding their design complexity, conditioning efficiency, power consumption, and they are usually designed for operation within a unique environment [35,36]. In this work, considering the application requirements, we use the combination of a simple bridge rectifier and a dynamic power management circuit to minimize the cost and maximize the efficiency and flexibility.

The changes in the power output from EHs result in variations in the amount of electrical energy gathered in the storage unit during the same period, making it difficult to accurately predict the performance of the EH after the WSN is already installed and in use. We design and present a dynamic power management scheme in this work where the behaviour of the circuits and WSN is adjusted and regulated according to the performance of the EH. The efficiency and effectiveness of the sensor node can be considerably improved compared with other schemes [37].

Most EHs including the two used in this work are only able to provide a very small amount of output power on the order of 10 μW to 100 μW [38,39,40] at an electricity usage level from a few amps to a few tens of amps, which puts a constraint on the energy available to operate the control and communication units [41]. Therefore, the entire WSN is expected to be in proper motion under a substantially restricted power budget below milliwatts. Considering this restriction associated with the operation of EH units, a cold start circuit is necessary to regulate the unsteady and weak output of the EH.

### 2.3. Wireless MCU

The microcontroller is one of the most fundamental and important elements in the operation of wireless sensor nodes and it is responsible for consuming the main portion of the total energy available to a single node. Thus, it is essential for the MCU to sustain a high level of functionality and performance while simultaneously minimizing the energy consumption. Selecting the proper MCU for a particular application is mainly dictated by factors such as computational requirements of the sensor node, power consumption, size, cost, and reliability.

Through a detailed and comprehensive investigation of our application requirements, the CC2640R2F Wireless MCU (Texas Instruments, Dallas, TX, USA) is selected to complete the operation of analog-digital converter (ADC) and data transmission. Ultra-low power consumption, the availability of development tools and libraries, ease of integration with the EH, and the available on-board wireless technology are the important factors to make it the most suitable candidate for our WSN system. The CC2640R2F Wireless MCU contains an onboard 2.4-GHz RF Transceiver that supports Bluetooth low energy (BLE) connectivity [42]. The unit’s BLE controller and its host libraries are embedded in the read only memory (ROM) which improves the overall power consumption of the unit. The CC2640R2F can be manipulated under several power modes (active, idle, standby, shutdown), making designers utilize the restrictive energy collected by the low-power harvesters more effectively. By programming the MCU according to the requirements of our intended application, we further enhance the efficiency of the system by disabling functions available in the MCU that are not necessary. Additionally, the MCU has an internal DC-DC converter that we fully utilize as a voltage regulator, enabling the power consumption efficiency of the unit to be further enhanced. The on-chip DC-DC converter powers the MCU as long as its input voltage is within the operational range of the device, which serves as another essential reason that we choose this device for energy-harvesting applications.

## 3. Hardware and Software Design

As previously shown, the power output of the EH is a function of the current intensity in the conducting wire and is susceptible to the variations in the current. As a consequence, the rectified output is unstable to provide a power supply to the WSN system. In addition, some inevitable power outages could completely disrupt the operation of the EH, which may result in a system shutdown. It is important that the system can automatically recover after the power outage. The practical interface circuitry must be compatible with both the EH and the control and communication unit while being functional over a wide range of operating conditions. Meanwhile, the MCU is supposed to complete operations (in active mode) to interact with the WSN most effectively within the shortest time to lower the limited power budget. These above anticipations have been realized by (1) designing and developing a dynamic power management interface circuitry and (2) developing and implementing embedded software algorithm used to operate the Wireless MCU for controlling the wireless sensor node’s operation, i.e., gathering, analyzing, and transmitting the sensory information to a central hub for post-processing purposes.

### 3.1. Circuit Design

**Figure 6:**
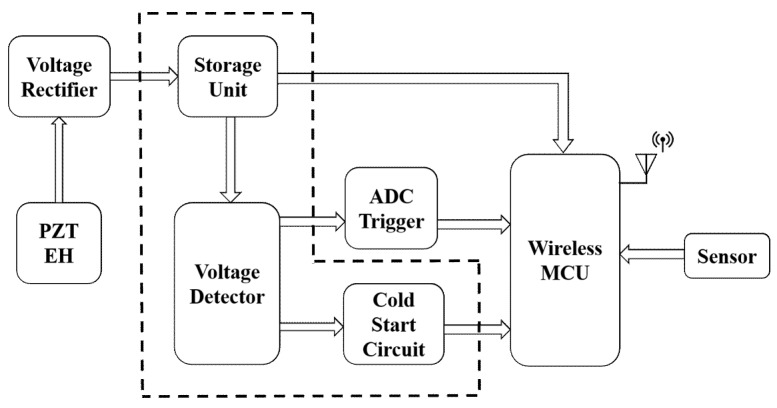
WSN system design architecture of a single sensor node. The arrows show the directions of different signals passing on within the system. The dashed box indicates the power conditioning module including storage unit, voltage detector, and cold start circuit.

Figure 6 presents the WSN system design architecture (the arrows show the directions of different signals passing on within the system) of the proposed single sensor node, where the power conditioning module is shown encompassed within the dashed outline. In the proposed design architecture, the voltage detector within the power conditioning module plays a significant role in the system’s behavior as it directly regulates the MCU’s operation by triggering the ADC. The triggering signal delivered by the voltage detector in turn adjusts the operating frequency of the wireless sensor node to transmit data packet on the basis of the performance of the EH. (Any input voltage within the range of 1.8 V to 3.8 V connected to the VDDS pin of the CC2640R2F effectively powers the unit.)

**Figure 7:**
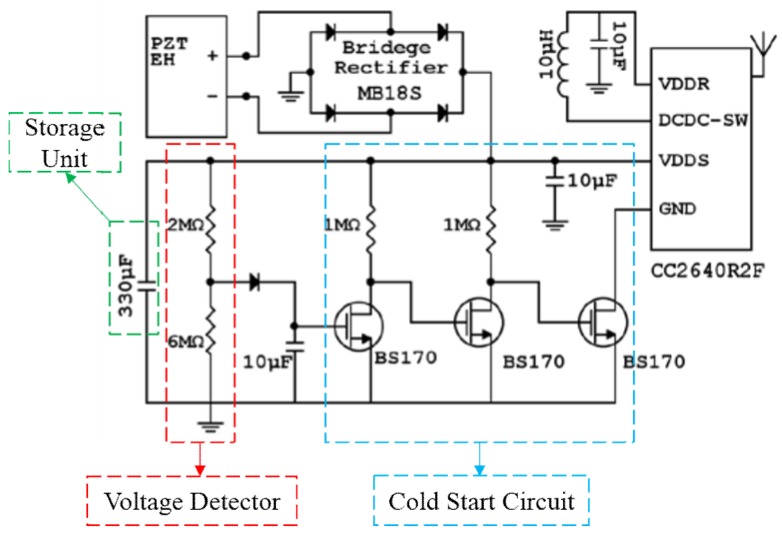
Detailed circuit schematic of the power conditioning module. The voltage detector detects the triggering information by actively monitoring the charging voltage across the storage unit. Once this voltage approaches the circuit threshold (3 V in this circuit), the circuit generates a trigger signal activing the Wireless MCU by starting the cold start circuit.

Figure 7 shows the detailed circuit schematics of the power conditioning module, with storage unit, voltage detector, and cold start circuit encompassed in the dashed boxes. A 330 μF capacitor is chosen to be the storage unit which is suitable to store enough energy for one and only one single data measurement and transmission activity. During the capacitor’s charging process, due to the connection between the ground pin of the MCU and the drain of the far right NMOS transistor which works in OFF region, the onboard ground node (GND) of the wireless MCU is disconnected from the real ground (i.e., GND is floating) so that the MCU is always shut down before one threshold point. This scheme suppresses any unintended and nonideal current leakage from MCU and allows the storage unit to be continuously charged/recharged before the voltage on the storage capacitor approaches the threshold. During the charging/recharging periods, the CC2640R2F MCU remains disabled.

Meanwhile, the output of the voltage divider (a 2 MΩ resistor and a 6 MΩ resistor) is delivered directly to the gate of the far left transistor with the addition of a small 10 μF capacitor to stabilize the node. As the voltage across the storage unit reaches the circuit threshold voltage, 3 V in this circuit, the voltage detector detects this triggering information by turning on the far left transistor. As a consequence, the output of the detector triggers the cold start circuit with a multistage amplifier and turns the far right transistor on, abruptly activating the CC2640R2F by reconnecting the GND pin of the MCU to the real ground node. At this time, the MCU will turn on and follow its instructions to complete the operations of measurement and data communication. It is worth mentioning that the simple circuit does not consider about different impedance from different EHs. On one hand, the simple circuit is able to work with the two different designs at a wide current range with duty cycle from <1 min up to 18 min, acceptable for the target application. On the other hand, one of the main application requirements is to design a cheap and robust system with flexibility to work with different EHs without adjusting the circuit.

### 3.2. Embedded Software Development

**Figure 8:**
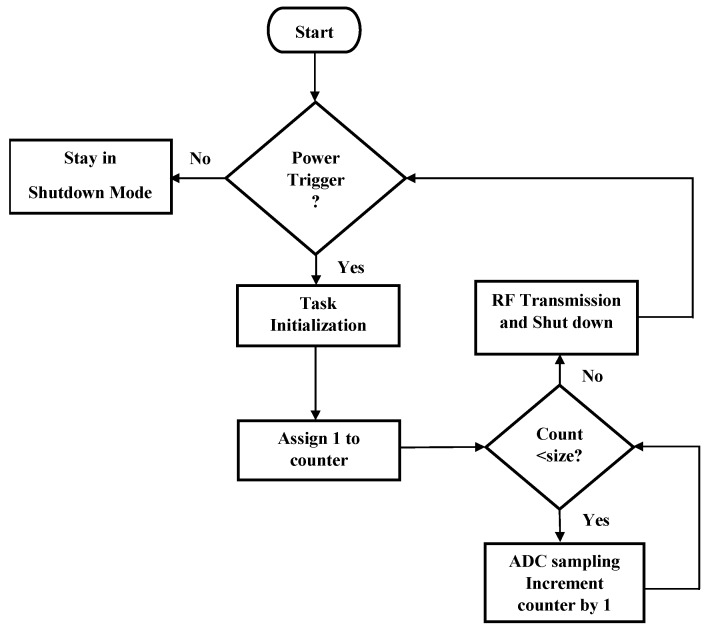
Wireless MCU process flow chart. Algorithm used to operate the Wireless MCU designed for controlling the wireless sensor node’s operation.

The MCU must be programmed according to the application requirements in order for the wireless sensor node to undertake function properly. Figure 8 shows the application flow chart diagram and algorithm developed for running the system and controlling the wireless sensor node’s operation. The process begins by enabling the MCU’s active mode through a trigger generated by the voltage detector. At this point, task initialization including the following steps begins:

- Clearing all the registers
- Pin initialization
- Turning on the GPIO Module
- Selection of the ADC input
- Scheduling the first task execution
- Setting up the ADC parameters
- Setting up the Radio parameters
- Requesting access to ADC
- Requesting access to the Radio

Once task initialization is completed, the ADC module starts to sample the input signal generated from the sensor device by obtaining a finite number of samples that are equidistant (with a constant sampling frequency) to cover one complete period of the alternating signal. Since the ADC’s lower bound of the operation voltage of this MCU is −0.3 V, the program instructs the ADC to only register samples from the positive portion of the sensor signal. Lastly, upon finishing collecting desired signal samples instructed by the program, the MCU creates data packets and the onboard RF module transmits the sampling information to the receiver hub for post-processing. After these operations, all the on-chip modules will be disabled and the MCU device will remain in a complete shutdown mode to furthest manage the small amount of energy harvested for the system until another power trigger signal arrives.

## 4. Implementation and Testing

To validate the circuit’s behavior and the performance of the self-contained integrated WSN, the system is tested according to the application requirements. Numerous different testing scenarios are developed to ensure the anticipated performance of the system under all conditions. Additionally, various stress tests are performed in order to confirm the sensor node’s stability under abnormal conditions including power outages, disruptions or rapid changes in the current passing through the wire, or loss of connection with the central hub.

**Figure 9:**
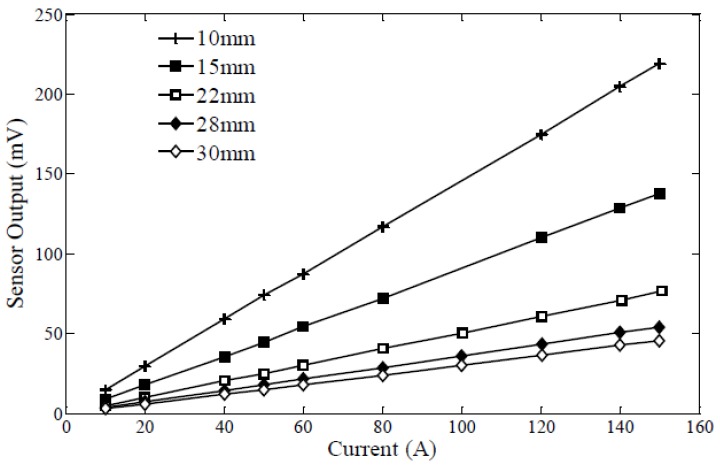
Sensor’s output (RMS voltage) with regard to the current passing through the wire (Output voltage is linearly proportional to the current intensity). Different plots represent different distances between the wire and the sensor [43].

The EH is mounted on an electric wire, mimicking the electricity grid in the residential/commercial buildings. A combination of several common appliances (such as a space heater and a hair drier) is used to draw current through the wire during testing at different levels between 5 A to 30 A. The current intensity in the electric wire is measured using an AC clamp-on meter (i400 s A Current Clamp, Fluke, Everett, WA, USA) separately before EH starts to harvest energy. As introduced in the previous section, the distance between EH1 and the wire is 6 mm and EH2 harvests energy from the wire at a distance of 4.5 mm for a higher power output. (Since the amplitude of vibration of EH1 is reasonably greater than EH2’s, EH1 is mounted relatively farther above the wire so as to protect it from permanent damage during vibration and also to decrease the nonlinear effects when the distance between the magnet and the wire is small.) The sensor chosen for this project was designed at the University of Waterloo’s Energy Harvesting Laboratory [43] and its output (converted from AC peak-to-peak to their corresponding Root Mean Square (RMS) values) is linearly proportional to the current intensity in the wire (shown in Figure 9). During testing, this sensor is placed approximately 10 mm above the wire with its outputs connected to the designated pins (ADC input) of the MCU so as to be sampled. A TDS 2114C oscilloscope (Tektronix, Beaverton, OR, USA) is used to monitor the signals from sensor output (sinusoidal signal) and circuit nodes.

Furthermore, in the central monitoring system, a CC2650 Wireless MCU LaunchPad™ Kit (TI’s SimpleLink™ Ultra-Low Power Portfolio, Texas Instruments, Dallas, TX, USA) is employed to receive the transmitted data packets and to validate the data transmission process. The receiver is connected to a desktop computer (as the central hub) using its onboard emulator and USB serial port. The hub is at a distance of one meter (within maximum distance around 100 m supported by the BLE 4.2 specification) away from the sensor node.

## 5. Results and Discussion

Once the unit is mounted in place, sensor data is transmitted to the receiver hub by the wireless MCU in order to validate the sensor node’s operation. Moreover, key performance parameters including the initial charging time, recharging time, operating frequency, and duty cycle are presented and their dependency to the EH’s performance is investigated. Comparative analysis of circuit behavior using different EHs is also illustrated. Lastly, stress tests are performed to verify the reliability of the system.

### 5.1. Performance Validation

**Figure 10:**
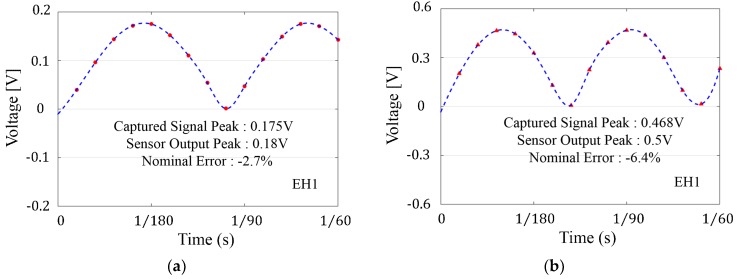
(**a**) Captured ADC values for a 60 Hz, 0.36 V peak-peak signal (**b**) Captured ADC values for a 60 Hz, 1.0 V peak-peak signal. Transmitted ADC values (red dots) for two arbitrary signal inputs to the wireless sensor node (with 15 positive points representing a full cycle of the sensor’s sinusoidal signal). The blue dashed curve shows the fitting result of the sensor’s signal with absolute values.

In order to generate data packets to represent a full cycle of the sensors’ signal during each data transmission to decrease the error rate and to verify the consistency and reliability of the device’s operation over time, multiple full-wave rectified sinusoidal signals replicating the sensor’s output for different experiments are fed into the sensor node and the transmitted ADC values are captured and compared to the peak voltage values of the input signal. In Figure 10, the captured data points for two of the arbitrary signals at the receiver node are plotted of a read and transmit process (with 15 points representing a full cycle of the sensor’s signal). The nominal errors associated with peak measurement are −2.7% and −6.4% respectively as a result of discrete sampling, which are caused by the limited number of points available from discrete sampling to cover the sensor’s signal. The measurement results here validate the sensor node’s operation as the peak measurement errors are less than the calculated worst case error of −8.6% (i.e., with an accuracy of 91.4%) as a result of discrete sampling. (If one uses 15 equidistant points to represent one cycle (with period T) of a sinusoidal waveform, the best case is to collect the point with peak value as the measured data, ideally with zero error. Whereas the worst case happens when the point T/15 away from peak point is chosen to stand for the peak value).

### 5.2. Transmission Duty Cycle Analysis

An essential characteristic of self-contained wireless sensor nodes is their duty cycle or operating frequency. In the proposed design, the duty cycle (the frequency of operation) of the sensor node is directly regulated by the triggering signals provided by the voltage detector circuit introduced in Section 3.1, and is self-adjusted according to the output of the EH.

**Figure 11:**
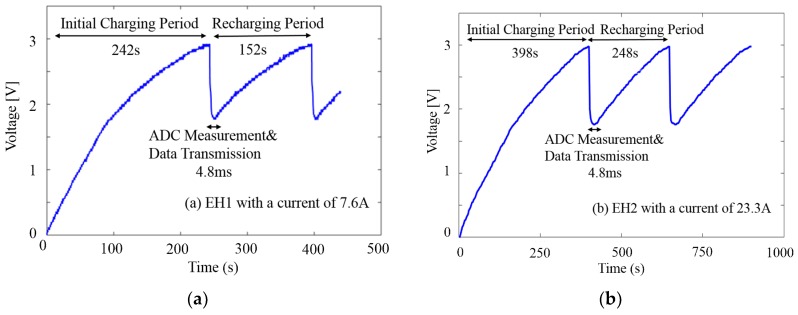
Charging/discharging behavior of the storage capacitor for WSN operations when the current in the wire is (**a**) 7.6 A (with EH1) and (**b**) 23.3 A (with EH2), respectively. In the beginning, it needs an initial charging period to accumulate enough energy to complete its very first read and transmit operation. Upon completion of a single ADC read and transmit process, the voltage will drop abruptly to below 2 V. At this moment, the MCU returns to the Shutdown mode until it re-accumulates enough energy to send the consecutive data packet.

In the beginning, it needs an initial charging period to accumulate enough energy from none to complete its very first read and transmit operation. The dynamic behavior of the storage unit with EH1 harvesting from 7.6 A grid current is shown in Figure 11a. It takes 242 s for the storage capacitor to reach 3 V from 0 V to initiate the wireless MCU’s operation. Upon completion of a data sampling and transmission process instructed by the program, the voltage across the capacitor will drop abruptly to below 2 V due to the partial energy consumed by MCU’s operation (the total MCU active time for ADC measurement and wireless transmission is measured to be approximately 4.8 ms). At this moment, the MCU returns to the shutdown mode until it re-accumulates enough energy to send the consecutive data packet. Nevertheless, the relatively long charging time only appears in the very first period. As long as the initial charging process of the storage capacitor is completed, the EH only needs to make up for the voltage drop (around one third of the threshold voltage) caused by the data packing and transmission of the WSN, and the time between subsequent data transmission (recharging period) is much reduced. Figure 11b shows the similar dynamic charging/discharging behavior of the system when the WSN is integrated with EH2.

As the current intensity in the wire increases, the EH’s output power rises accordingly. Higher power supply produces same amount of electric charge/energy within a shorter period of time, implying a lesser charging time of the storage capacitor. Similarly, the recharging time for following processes will be reduced as well. In this way, the frequency of operation is dynamically adjusted to increase with the incremental current passing through the wire.

**Figure 12:**
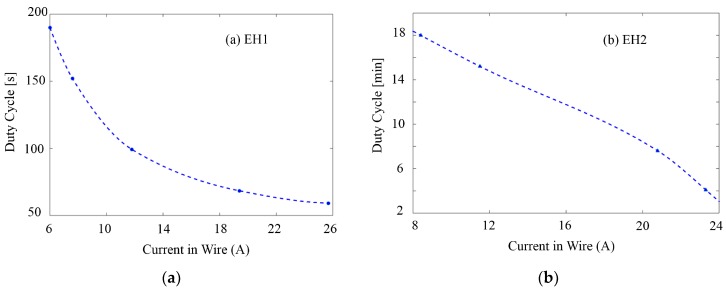
Duty cycle of operation with regard to current intensity passing through the wire when the system is tested with (**a**) EH1 and (**b**) EH2. Higher current indicates a shorter duty cycle because of a weaker output power supplied by the EHs.

Figure 12 presents the relationship between the duty cycle of the system and current intensity in the wire tested with EH1 and EH2 respectively. As expected, the duty cycle of operations is compressed by the increasing current intensity. The measurement with the EH1 demonstrates that the WSN system is capable of achieving a duty cycle from <1 min to 2.5 min when the current ranges from 7.6 A to 30 A, easily meeting the need in the applications of monitoring electricity usage in large buildings. The measurement results of EH2 indicate this system powered by the advanced weaker harvester is in a position to bring about duty cycle from <4 min to 18 min for a current varying from 8.4 A up to 30 A. In this case, the duty cycles become much longer than those of previous test due to the weaker output power supplied by the meandering PZT-based low-power harvester. In fact, if the current intensity continuously goes lower than 8 A, the power output of the EH2 will reach the system’s lower power supply limit at some point, i.e., the EH’s power output tends to be not powerful enough to charge and recharge the storage capacitor effectively because of the leakage current and static power dissipation of the system.

### 5.3. Reliability Verification

**Figure 13:**
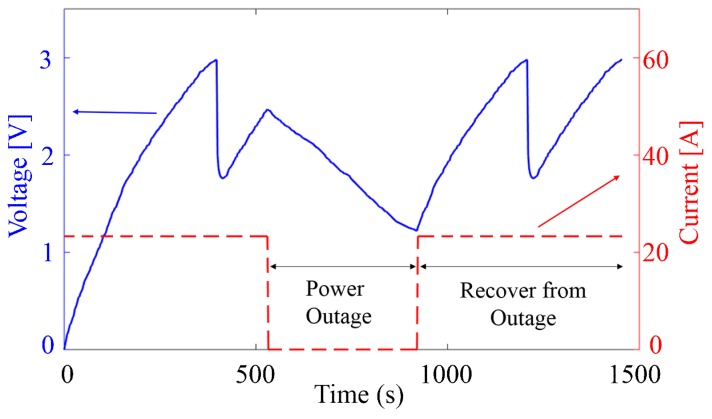
Charging/discharging behavior of the storage capacitor for WSN operations when a power outage occurs. Tested with EH2 when the current in the wire is 23.3 A before/after the outage. Successful completion of data transmission demonstrates the system’s automatic recovering from outages.

For the sake of further verifying the robustness and dependability of WSN system’s operation over time under various conditions, stress tests are designed and performed:

– The system is tested in the case of current intensity varying rapidly passing through the electric wire to validate its robustness. Experimental results show that the unit have the correct behavior under this abnormal circumstance and changing the current intensity just influences the duty cycle of the operation.
– The system is then tested under the circumstance of power outage. Figure 13 presents the system’s behavior when a power outage occurs during a recharging period. The storage unit starts charging again after the power outage is recovered. The desired data sample packets are successfully created and transmitted by the WSN in this accidental situation, demonstrating the system’s automatic recovering from outages.
– Other stress tests including disruptions in the current and the sudden disconnection with the central controlling hub are also performed so as to confirm that the self-powered system is able to recover from these abnormal conditions.

## 6. Conclusions

The design, development, and testing of a smart, low-power wireless sensor node system for real-time electricity monitoring applications within residential, educational, and commercial buildings are presented in this paper. By harvesting energy from the wire being monitored, the unit is battery-independent and is able to wirelessly gather, process and transmit the data packets sampled from the non-invasive sensor via Bluetooth connectivity with a low error less than 8.6%. Enabled by a dynamic power management scheme, the intelligent sensor node can self-adjust its duty cycle depending on the characteristics of different EHs and the current being monitored. Without any need of modification or adjustment, the circuit is able to operate with two different EHs at a wide range of input power and pass different stress tests, proving the flexibility and robustness of the system. This simple and state-of-the-art integrated system serves as a promising solution to the target applications.
